# Cognitive behavioral therapy for ADHD predominantly inattentive presentation: randomized controlled trial of two psychological treatments

**DOI:** 10.3389/fpsyt.2025.1564506

**Published:** 2025-04-10

**Authors:** Elinor Eskilsson Strålin, Lisa B. Thorell, Tobias Lundgren, Sven Bölte, Benjamin Bohman

**Affiliations:** ^1^ Department of Clinical Neuroscience, Centre for Psychiatry Research, Karolinska Institutet, & Stockholm Health Care Services, Region Stockholm, Stockholm, Sweden; ^2^ Department of Clinical Neuroscience, Karolinska Institutet, Stockholm, Sweden; ^3^ Department of Women’s and Children’s Health, Karolinska Institutet, Stockholm, Sweden; ^4^ Child and Adolescent Psychiatry, Stockholm Health Care Services, Region Stockholm, Stockholm, Sweden; ^5^ Curtin Autism Research Group, Curtin School of Allied Health, Curtin University, Perth, WA, Australia

**Keywords:** ADHD, ADHD predominantly inattentive presentation, CBT, group intervention, behavioral activation, randomized controlled (clinical) trial

## Abstract

**Introduction:**

Attention-Deficit Hyperactivity Disorder (ADHD) in adults is common and characterized by difficulties in regulation of attention, activity and goal directed behaviors. These challenges are especially associated with inattentive symptoms, why high levels of inattention imply severe impairment in everyday life. CBT for ADHD-inattentive presentation, (CADDI), is designed to treat inattention and incorporates skills training in organization, behavioral activation, and mindfulness. The aim of this study was to compare the effectiveness of CADDI with regular CBT treatment for ADHD.

**Methods:**

A multicenter, pragmatic, two-arm, parallel, randomized controlled trial compared CADDI to Hesslinger’s dialectical behavior therapy protocol. The study included 108 participants from six psychiatric outpatient clinics in Stockholm. Self-reported scales were used to assess outcome measures of behavioral activation, procrastination, symptoms of ADHD, depression, quality of life and functional impairment. Data were analyzed for between and within-group effects using multilevel modeling.

**Results:**

Between-group analysis showed that participants in the CADDI group had significantly greater improvement on the primary outcome measure of activation at post assessment (*p* = .045, *d* = 0.49). No significant between-group effect was found on the other measures. However, within-group analysis showed larger effect sizes in the CADDI condition relative to the control condition on several measures. Adherence was good and attrition 21.3% despite effects of the pandemic. Participants and therapists reported higher satisfaction with CADDI as compared to the control group.

**Discussion:**

This trial demonstrated that CADDI was more effective regarding behavioral activation and suggests a potential advantage of an intervention specifically targeting ADHD-I over generic CBT for ADHD. However, the trial was underpowered and failed to prove between-group effects in spite of large differences in effect sizes on several measures. Future research with larger samples and long-term follow-ups is recommended to validate and expand upon these results.

## Introduction

Attention-Deficit/Hyperactivity Disorder (ADHD) is a persistent and heterogeneous neurodevelopmental condition operationalized by three phenotypic presentations in DSM-5 and ICD-11: predominantly inattentive (ADHD-I), predominantly hyperactive/impulsive (ADHD-H), and combined presentation (ADHD-C) (1, 2). ADHD is common, affecting 5 to 6% of youth and 2 to 3% of the adult population (3) and about 6-7% report impairing symptoms of ADHD in adulthood (4). In adults, ADHD is linked to difficulties in task and time management, self-regulation of behavior and affect regulation (5, 6). These challenges contribute to behaviors such as procrastination and inconsistency in task completion (7, 8). ADHD in adults is associated with negative outcomes in several domains, such as lower educational attainment, subsequently leading to reduced employment opportunities, lower income, and higher rates of sick leave (3, 5, 9, 10). Adults with ADHD are also more likely to suffer from comorbid psychiatric disorders and somatic conditions, leading to adverse outcomes regarding health, socioeconomic status and shorter life expectancy (3).

Multimodal treatment including psychoeducation, pharmacotherapy, and Cognitive-Behavioral Therapy (CBT) is recommended for adult ADHD (5). CBT is administered in structured treatment protocols, focusing on the acquisition of compensatory skills to cope with common difficulties in ADHD. Meta-analyses of CBT for adult ADHD (11–13) show reductions in ADHD symptoms of medium-to-large effect sizes in comparisons with waitlist control groups and small effect sizes favoring CBT in comparison with active control conditions (i.e., psychoeducation, supportive therapy, clinical management, relaxation; (14). The Hesslinger protocol is an adaptation of dialectical behavior therapy to suit ADHD and is designed for treatment in a group format (15). The protocol is widely used and features mindfulness and behavior analysis along with psychoeducation on themes related to ADHD. This therapy has proven feasible in clinical settings, reducing symptoms of ADHD, and is considered effective by participants (16–20). However, the protocol focuses less on organizational skills, which is a common problem area causing many everyday challenges in adult ADHD. CBT protocols addressing organizational difficulties in adults with ADHD have been developed in both individual (21, 22) and in group format (23). These protocols target time-management, planning, and distractibility, and have proven effective in reducing ADHD symptoms in comparison to active control conditions. However, these protocols do not include mindfulness, which is why some authors (24, 25) have proposed combining components of organizational skills from CBT protocols (21, 23) and components of mindfulness from the Hesslinger protocol (26). However, a potential limitation of existing CBT protocols for ADHD is that they are generic to ADHD, and thus do not consider that there are three rather dissimilar presentations characterized by different core symptomatology.

ADHD is characterized by challenges in higher-order cognitive processes such as planning, organization, initiation, inhibition, shifting, emotional control, and working memory (27–29), these cognitive challenges are especially associated with inattentive symptoms (6, 30). Inattention is more associated with impairment in adult life compared with hyperactivity/impulsivity (9, 30, 31). In addition, inattention is strongly related to increased levels of stress (32), difficulties in emotion regulation (33) and difficulties in initiating goal-directed behavior and achieve long term goals (34, 35). Furthermore, inattention is highly associated with academic and occupational underachievement and serves as a robust predictor of long-term impairment (9). These observations support the conclusion by Vitola et al. (30) that the adult ADHD phenotype is primarily characterized by inattentive symptoms.

CBT for inattention and associated problem areas ought to be comprehensive and include strategies to manage initiation difficulties and procrastination. The CBT for ADHD-I (CADDI) protocol was developed in a clinical setting where individuals with ADHD-I sought help for passivity and procrastination, expressing dissatisfaction with general CBT offered to all kinds of ADHD. CADDI was designed as a presentation-specific treatment for ADHD-I, incorporating skills training in organization, behavioral activation strategies, and mindfulness practice to reduce inattention and related difficulties. The CADDI protocol was inspired by the work on ADHD by Safren et al. (21) and Hesslinger et al. (26), and behavioral activation as described by Addis and Martell (36). In CADDI, behavioral activation is emphasized through strategies and skills training on how to implement planned activities, on initiation and termination of tasks. While initiating difficulties and procrastination are not symptoms defining ADHD, they are legitimate treatment targets, as they are behavioral consequences of inattention and lead to functional impairment. If difficulties in self-regulation of behavior are decreased, an increase in functional ability and quality of life may be expected. So far, no studies of CBT for ADHD have measured effects on activation or procrastination, although these behavioral difficulties are highly present in ADHD (34). The CADDI protocol has been previously tested in an open feasibility study (n = 39) and the results showed good feasibility, acceptability, and preliminary effects regarding inattention and depression symptoms (37). The CADDI protocol has also been investigated in a qualitative interview study, inquiring on participants’ experiences of treatment according to the protocol (38). Participants described getting increased understanding and acceptance of their condition and reported the practice of mindfulness to enhance attention. Further, participants emphasized the group setting as a facilitator of therapeutic effects (38).

The present randomized controlled trial aimed to evaluate the effectiveness of the CADDI protocol as compared to the Hesslinger protocol in adults with ADHD-I seeking psychological treatment in routine psychiatric services. First, we hypothesized that the CADDI protocol would generate greater change in terms of behavioral activation and procrastination as these areas are in focus in CADDI, but not specifically addressed in the Hesslinger protocol. Second, we also wanted to investigate whether the CADDI protocol would be superior to the Hesslinger protocol on the secondary outcome measures of depressive symptoms, functional impairment, and quality of life. Regarding symptoms of ADHD, we did not expect CADDI to be superior in reducing symptoms of inattention and hyperactivity, considering that the control condition was another CBT for ADHD.

## Method

### Design and setting

A prospective, pragmatic, two-arm parallel group randomized controlled multicenter superiority trial was conducted comparing the CADDI protocol with the Hesslinger protocol. The study used a 2:1 randomized controlled design, assigning two participants to the CADDI condition and one to the Hesslinger condition in every block of three. This was done in order to let participants randomized to the control condition receive treatment in groups including both study participants and other patients at the clinic diagnosed with ADHD-C or ADHD-H, to be close to treatment as usual. Participants were recruited from six psychiatric outpatient clinics in Stockholm, Sweden, from 2019 to 2023. Four clinics were public and two were private but had publicly funded care agreements. The study was approved by the Swedish Ethical Review Authority (2019-02444) and registered at ClinicalTrials.gov (NCT04090983).

### Participants and sample size

**Figure 1:**
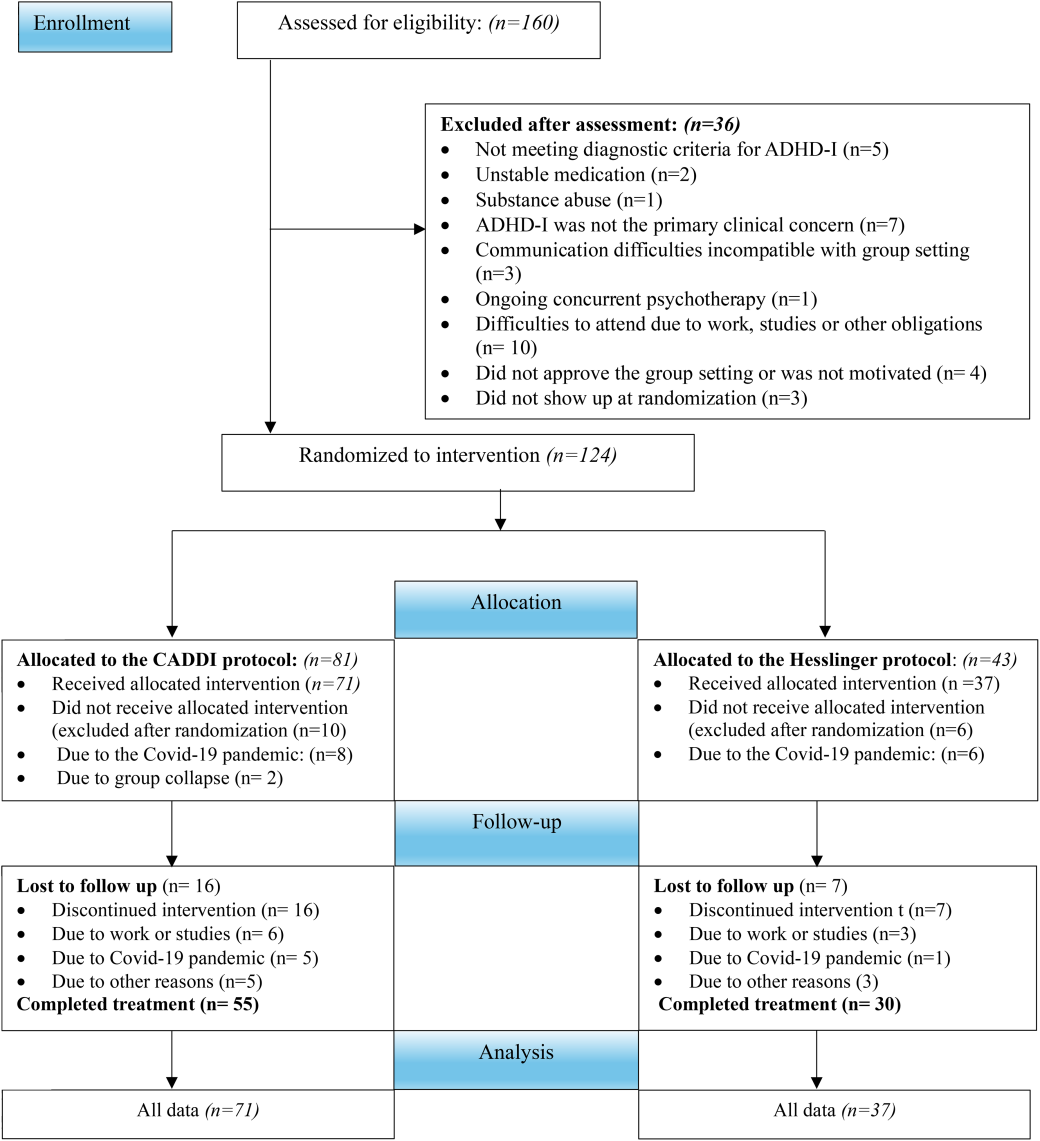
ADHD-I, Attention-Deficit Hyperactivity Disorder, inattentive presentation. Participants who were excluded after randomization due to the Covid-19 pandemic were participants who were randomized to and started treatment but could not complete treatment due to restrictions in terms of group meetings being cancelled. Participants who were excluded after randomization due to group collapse were participants who were not provided with the opportunity to complete treatment due to dropout of other group members and thus dissolution of the group format. “Covid-19 pandemic-related drop out” refers to participants who dropped out due to fear of transmission of disease or being contagious, and other pandemic-related issues.

In total, 108 participants were included in the study. They were recruited from psychiatric outpatient clinics located in different geographical areas of Stockholm (city, suburb), representing a variety of socioeconomic conditions. Inclusion criteria were: (a) a principal diagnosis of ADHD-I, (b) 18 years of age or above, (c) no change in medication in the last two months, and (d) completion of a psychoeducational course on ADHD regarding symptoms, self-care, and treatments. Exclusion criteria were: (a) diagnosis of intellectual disability, (b) substance abuse, (c) severe mental illness (e.g., severe depression, anorexia) or comorbidity requiring clinical priority, (d) difficulties in compliance with medical or other treatment, (e) difficulties allocating time to participate due to social, academic or occupational circumstances hindering engagement in treatment, (g) other ongoing psychotherapy, and (f) difficulties communicating, or accepting the group setting. There was no exclusion of participants with comorbid psychiatric diagnoses other than those mentioned above. For participant flow through the study, see **Figure 1**. Participants had a mean age of 36.4 years, 61.1% were female, 38.9% were male and they were diagnosed with ADHD-I at a mean age of 32.7 year. For further sociodemographic and clinical characteristics of participants at pre-assessment, see **Table 1**.

**Table 1:** Sociodemographic and clinical characteristics of participants at pre-assessment.

		CADDI (*n* = 71)	Hesslinger (*n* =37)	Total (*n* = 108)
Gender	Female n (%)	45 (63.4)	21 (56.8)	66 (61.1)
Age	Mean (SD) Range	36.0 (9.8) 20–66	37.2 (10.8) 24–64	36.4 (10.1) 20–66
Relationship status				
	Single	34 (47.9)	16 (43.2)	50 (46.3)
	Married, engaged or cohabiting	36 (50.7)	21 (56.8)	57 (52.8)
	Other kind of relationship	1 (1.4)	0	1 (0.9)
Education		n (%)		
	Elementary school	9 (12.7)	3 (8.1)	12 (11.1)
	High school	41 (57.7)	26 (70.3)	67 (62.0)
	College/University	21 (29.6)	8 (21.6)	29 (26.9)
Residence				
	Single household	32 (45.1	19 (51.4)	51 (47.2)
	Cohabiting	26 (36.6	17 (45.9)	43 (39.8)
	Living housed in someone else’s residence	13 (18.3)	1 (2.7)	14 (13.0)
Occupational status				
	Working	35 (49.3)	21 (56.8)	56 (51.9)
	Studying	11 (15.5)	4 (10.8)	15 (13.9)
	Unemployed	10 (14.1)	6 (16.2)	16 (14.8)
	Sick leave full time	6 (8.5)	1 (2.7)	7 (6.5)
	Sick leave part time	5 (7.0)	1 (2.7)	6 (5.6)
	Disability pension	3 (4.2)	1 (2.7)	4 (3.7)
	Parental leave	1 (1.4)	3 (8.1)	4 (3.7)
Income				
	Income from work or studies	44 (62.0)	23 (62.2)	67 (62)
	Unemployment insurance or supported employment	3 (4.2)	1 (2.7)	4 (3.7)
	Insurance fund for sick leave, parental leave	16 (22.5)	7 (18.9)	23 (21.3)
	Social assistance	2 (2.8)	0	2 (1.9)
	No income	6 (8.5)	6 (16.6)	12 (11.1)
Support in everyday living^a,b^				
	Support from relatives	0	8 (21.6)	
	Community based support	0	5 (13.5)	
Age when diagnosed with ADHD-I^c,d^		32.4 (11.0)	33.3 (12.4)	32.7 (11.5)
Other NDD				
	Dyslexia	3 (4.2)	5 (13.5)	8 (7.4)
	ASD	11 (15.5)	3 (8.1)	14 (13.0)
Psychotropic medication^e^				
	ADHD medication only	18 (25.4)	17 (45.9)	35 (32.4)
	Other psychotropic medication only	12 (16.9)	0	12 (11.1)
	Both ADHD and other psychotropic medication	26 (36.6)	17 (45.9)	43 (39.8)
	No psychotropic medication	14 (19.7)	3 (8.1)	17 (15.7)
Previous psychotherapy^f,g^				
	CBT only	18 (25.4)	7 (18.9)	25 (23.1)
	Other psychotherapy only	18 (25.4)	9 (24.3)	27 (25.0)
	Both CBT and other psychotherapy	24 (33.8)	10 (27.0)	34 (31.5)
	No previous psychotherapy	6 (8.5)	8 (21.6)	14 (13)

ADHD-I, attention deficit hyperactivity disorder, predominantly inattentive; ASD, Autism spectrum disorder; CBT, cognitive-behavioral therapy; NDD, Neurodevelopmental disorder.

^a^Missing: CADDI, *n* = 1, ^b^Missing: Hesslinger, *n* = 1, ^c^Missing: CADDI, *n* = 8, ^d^Missing: Hesslinger, *n* = 4,

^e^Missing: CADDI, *n* = 1 ^f^Missing: CADDI, *n* = 5, ^g^Missing: Hesslinger, *n* = 3.

Because no previous study has examined differences between two psychological treatments for ADHD, there was no empirical basis for sample size calculation. However, it was expected that the focus on activity initiation and procrastination in the CADDI protocol would result in markedly superior effects on the primary outcome measures. To detect a between-group difference of Cohen’s d = .50, with power of .80 and p <.05 (two-tailed test), on the primary outcome measure of activation (Behavioral Activation for depression short form, BADS-SF, (39) a sample size of 144 participants was required, taking account of the unbalanced design and allowing for 25% dropout. Data collection in this study was affected by the COVID-19 pandemic starting in March 2020, as measures taken to prevent transmission included restrictions on group treatments. Fourteen participants did not receive treatment as randomized due to the sudden pause in group treatments in March 2020 and were excluded from the study. Group therapy was resumed later in 2020, but during 2020-2021, the threat from the pandemic fluctuated with the seasons, leading to restrictions on group sizes, social distancing, and the wearing of protective facial masks. These circumstances disrupted ordinary interaction in groups and caused some dropout among participants. For instance, when the pandemic incidence rate increased, some participants stopped coming due to being contagious or fear of transmission. The Covid-19 pandemic hindered the data collection and when restrictions due to the pandemic ceased, many caregivers had less resources and were hesitant to engage in research. The recruitment phase of this study was prolonged to increase sample size but could not be continued further than 2023.

### Treatments

#### The CADDI protocol

The CADDI protocol was developed by the first author together with her clinical colleagues to meet the needs of patients with ADHD-I in psychiatric outpatient care. The protocol was developed to be delivered in a group format with individual follow-up, to provide support to home assignments in treatment. Beside the focus on organizational skills and activation, the CADDI protocol uses mindfulness practice as defined by Bishop et al. (40). Mindfulness is practiced to enhance self-regulation of attention, which involves direction of focus, sustaining, and switching of attention as well as inhibition of elaborate processing. Practice of mindfulness has proven to reduce symptoms of ADHD (41, 42) and executive dysfunction in adults with ADHD (43, 44). Mindfulness meditation also entails increased awareness of emotional states and is a tool for emotional regulation (45). Difficulties with initiating activity is addressed through strategies from behavioral activation (BA), an intervention developed to treat depression through increasing activity (36). In BA, passivity is addressed through strengthening the capacity to engage in activities despite depressed mood. The strategies for dealing with avoidance of negative emotions in BA along with hands-on strategies for procrastination may be useful interventions regarding passivity and procrastination in ADHD-I. Behavioral analysis is used in sessions to identify reinforcement contingencies.

The CADDI protocol was structured to provide support to overcome difficulties with inattention that might interfere with treatment. Therefore, home assignments are initiated in session, and participants are encouraged to share the content of each session with a significant other to enhance learning and involve close ones in the treatment. Furthermore, all major components in the protocol are rehearsed over two sessions and followed up continuously in the group. Some sessions are dedicated solely to repetition to enhance the acquisition of new habits and routines. To support retention of treatment gains after its termination, the last three sessions are devoted to making a maintenance plan. Participants received handouts of presentations and worksheets both in paper form and digitally via a weblink. The content of the CADDI protocol is presented in a brief overview in **Table 2**.

**Table 2:** Content of the CBT for ADHD-I (CADDI) protocol and the Hesslinger protocol.

CADDI		Hesslinger	
Session Content		Session Content	
1	Introduction to treatment Basic organizational tools	1	Introduction to treatment Psychoeducation on ADHD, Goals
2-5	Mindfulness in theory and practice	2	Neurobiology, Mindfulness
	Organizational tools: Using calendar, to-do list, prioritize, daily routines, divide big tasks into smaller pieces. Setting goals and defining action in accordance with goals.	3-4	Mindfulness in theory and practice
		5	Organization: difficulties and strategies
6-7	Behavior initiation: Skills for activity initiation, Weekly report chart to support use of strategies Behavior analysis	6-7	Behavior Analysis: Understanding dysfunctional behavior and how to change
8-9	Termination of activity: Strategies to complete and cease activity. Weekly report chart to support use of strategies Troubleshooting on activity initiation and termination Behavior analysis	8	Emotion regulation Behavior Analysis
		9	Depression/Medication Behavior Analysis
		10	Impulse control Behavior Analysis
10-11	Coping with stress Stress management strategies Weekly report chart to support use of strategies Behavior Analysis	11	Stress Management Behavior Analysis
		12	Dependency, Substance abuse Behavior Analysis
12 -14	Maintenance plan: Maintaining behavioral changes after therapy, managing setbacks, sticking with the maintenance plan.	13	ADHD in relationship and self -respect, Behavior Analysis
		14	Retrospect and Outlook

#### The Hesslinger protocol

Participants randomized to the control condition received treatment according to the manual developed by Hesslinger et al. (Hesslinger et al. (15) in its published Swedish translation (26). This protocol includes psychoeducation on ADHD, symptoms, comorbidities, and associated difficulties. The protocol features the theory and practice of mindfulness and behavioral analysis as tools to address behavior problems. The components of the Hesslinger protocol increases awareness of thoughts, emotions, and impulses, and strengthen goal-directed behavior as opposed to short-termism, thereby addressing common behavioral difficulties in ADHD. Home assignments include conducting behavioral analyses and practicing mindfulness. All group members received a copy of the treatment protocol and had access to material at the publisher’s website. The content of the Hesslinger protocol is presented in a brief overview in **Table 2**.

#### Group format

Both the intervention and the control condition were delivered in a group format consisting of 14 weekly 2-hour sessions, led by two group leaders. Each session followed a set agenda with recurring components according to the protocols, including mindfulness practice, a review of home assignments, a coffee break, mindfulness practice, presentation of themes, skills training, and planning of home assignments. In both treatments mindfulness meditation was practiced in session and as home assignments, both in exercises of staying present in everyday situations and through audio recordings of meditation exercises. After each session, an optional individual consultation (<30 minutes) was available for participants in need of extra support regarding the treatment. Between each session, a scheduled telephone call by a group leader supported adherence to home assignments and followed up on participants’ goals in treatment. During the pandemic the administration of the treatments were adapted and participants who were not able to participate in the group sessions for two weeks in a row were offered an individual session over telephone or video to be able to keep following the treatment and to prevent drop-out. Group sizes at treatment start ranged from 5 to 8 in the intervention condition (M=6.1, SD=1.1) and from 4 to 9 in the control condition (M=7.3, SD=1.7).

### Therapists, training, and supervision

Thirty-four therapists participated in the study: 23 (67.6%) were female, and 11 (32.4%) were male therapists, with a mean age of 38.0 years (SD=11.7). Thirty-one (91.1%) were psychologists, of whom 21 (67.7%) were licensed clinical psychologists, and 10 (32.3%) were psychology residents when they first became group leaders. Of the resident psychologists, two continued as group leaders after obtaining their licenses. Two therapists were social counsellors with psychotherapy training and one therapist was mental health worker trained in dialectic behavior therapy. The majority of therapists (32 individuals, 94.1%) were trained in CBT or DBT, while the remaining two had other psychotherapy training. Two of the psychologists and one of the social counsellors were licensed psychotherapists. At pre-treatment, therapists had on average 4.3 (SD= 4.9) years of experience working with ADHD in assessment, consultation or therapy, ranging from 0 to 20 years. Therapists had on average 3.6 (4.1) years of experience as therapists before entering the study, ranging from 0 to 15 years. Therapists had on average conducted 2.5 (3.4) CBT groups for ADHD using the Hesslinger protocol, ranging from 0 to 15 groups. Generally, groups were led by two group leaders, at least one of the leaders in each group was experienced in CBT. Therapists were not allowed to lead both CADDI and Hesslinger groups simultaneously, and former CADDI group leaders were not allowed to conduct Hesslinger groups to avoid contamination of treatment content in the control condition. On average, therapists conducted 1.4 (SD= 1.3) groups each, ranging from 1 to 7 groups.

Prior to treatment start, all therapists received a two-hour presentation covering CBT for ADHD and a one-hour instruction on how to conduct groups in the study by the first author. Group leaders in the intervention condition received an additional lecture on the CADDI protocol. All group leaders received supervision on three occasions for 60 minutes each, around session 4, 7 and 10 of each treatment period. Initially, eight pairs of group leaders in both conditions were supervised by the first author, however, half of these groups were canceled due to the pandemic. Thereafter, therapists in the control condition were supervised by an independent clinical psychologist, with an extensive experience in the Hesslinger protocol. Therapists in the CADDI protocol were supervised by the first author who was also active as a group leader in the study.

### Assessments

#### Eligibility measures

Diagnosis of ADHD-I was verified using the Diagnostic Interview for Adult ADHD, (DIVA; 46). DIVA serves to assess the presence of ADHD symptoms and functional impairment due to these symptoms through a clinical interview. Substance abuse was screened with the Alcohol Use Disorder Identification Test (AUDIT; 47) and the Drug Use Disorder Identification Test (DUDIT; 48). Both tests are self-report measures covering the quantity and frequency of substance intake, dependence, and adverse consequences. Patient records were used to decide on the exclusion criteria of intellectual disability and severe mental disorder.

#### Primary outcome measures

Two instruments measuring activation and procrastination were used as primary outcomes. The Behavioral Activation for Depression Scale Short Form (BADS-SF; 39) measures activation and contains 9 items scored from 0 (not at all) to 6 (very much). BADS-SF reflects a positive value for activation through the total score ranging from 0-54 and captures underlying dimensions through the subscales of Activation and Avoidance. BADS-SF has shown good internal consistency (α = .82) and construct validity, well correlated to other relevant measures as shown by Manos et al. (39). In the present study, internal consistency in the total sample at pre-treatment was α = 0.77. The Pure Procrastination Scale (PPS) is a 12-item scale developed by Steel (49) to capture procrastination as a dysfunctional delay of intended action. The PPS is scored from 1 (not at all) to 5 (very much). The PPS has shown excellent psychometric properties (α = .92) and convergent validity with other related measures (49). In this study, the internal consistency of PPS was α = .89.

#### Secondary outcome measures

ADHD symptoms was assessed through the Adult ADHD Self Report Scale (ASRS; 50). The ASRS comprises18-items corresponding to the ADHD symptoms found in the DSM measuring frequency and are rated from 0 (never) to 4 (very often). The ASRS has good psychometric properties (α = .88) and in the present study internal consistency in the total sample at pre-treatment was (α = .82). Inattention and associated difficulties were assessed using the Brown Attention Deficit Disorder Scales (BADDS; 34). The BADDS was developed based on the definition of Attention-Deficit Disorders (ADDs) as presented in DSM-IV and captures inattention and associated problem behaviors in five dimensions: Organizing and activating to work, Sustaining attention and concentration, Sustaining energy and effort, Managing affective interference and Utilizing working memory and Accessing recall. BADDS measure the frequency of symptoms and include 40 items rated on a 4-point scale, from 0 (never) to 3 (almost daily). The BADDS have shown excellent internal consistency (Cronbach’s α = 0.96; 34) and in this study the alpha level at pre-assessment was good (α = 0.88). Quality of life was assessed through the Adult Attention-Deficit/Hyperactivity Disorder Quality of Life Scale (AAQoL; 51). AAQoL is a 29-item scale scored from 1 (Not at all/Never) to 5 (Extremely/Very Often). AAQoL measures quality of life in four domains: Life Productivity, Psychological Health, Relationships, and Life Outlook. The AAQoL has shown an overall internal consistency of α = .93 (51), in this study was α = 0.88. Depressive symptoms were assessed using the Patient Health Questionnaire-9 (PHQ-9; 52), a nine items questionnaire, rated from 0 (almost never) to 3 (almost daily). The PHQ-9 has shown internal consistency of α = 0.89 (52) and in this study it was α = 0.84 at pre-assessment. Functional impairment was assessed using the World Health Organization Disability Assessment Schedule, 12-item version, (WHODAS; 53), rated from 0 (no difficulty) to 4 (extreme difficulty). WHODAS has a high internal consistency of α = 0.98, in this study it was α = 0.83 at pre-assessment. Mindful awareness was assessed using the Mindful Attention Awareness Scale (MAAS; 54). The MAAS assesses characteristics of mindfulness, through 15 items rated from 1 (almost always) to 6 (almost never). The MAAS has shown good internal reliability, (α = 0.89, MacKillop and Anderson (55), in the present study, internal consistency in the total sample at pre-treatment was α = 0.85.

#### Other measures

Participants’ adherence to home assignments was estimated by therapists at session 4, 7, and 10 using a single item ranging from 1 (participant did not try to do home assignments) to 6 (participant did more home assignments than required), with intermediate scores describing different levels of completion. Therapists’ adherence to the treatment protocols was measured through self-assessment around session 4, 7 and 10 during treatment, using a questionnaire developed for the purpose of this study. Through nine items, the questionnaire asked the therapists, to what extent they had implemented the agenda and intent of the protocol at the most recent session (e.g. “Each participant had the opportunity to report his/her home assignment and get feedback on it”, “The theme of the session was presented”, “Participants’ understanding of the theme was ensured”). The items were rated; 0 (not at all), 1 (to a low degree), 2 (to a medium degree), 3 (to a high degree), 4 (to a very high degree).

The treatments were evaluated by participants using the Client Satisfaction Questionnaire-8 (CSQ-8; 56) containing eight questions scored on from 1 (low satisfaction) to 4 (high satisfaction), showing an internal consistency of α = 0.92. The usability of the treatment protocols was evaluated by the therapists at the end of treatment. The therapists’ evaluation form was constructed for this study and contained nine questions regarding the relevancy, comprehensibility and usefulness of the protocol regarding the needs of participants (e.g. “The content of the protocol is relevant regarding the symptoms it is intended to treat”, “The interventions in the protocol correspond to the needs of participants”). The items were rated; 0 (not at all), 1(to a low degree), 2 (to a medium degree), 3 (to a high degree) 4 (to a very high degree).

### Procedure

Eligible participants were informed about the study by clinic staff, through posters in waiting rooms, information available in media covering ADHD research and clinic websites. All participants were given oral and written information on the study procedures, including randomization and the control condition, and provided their informed written consent. Prior to inclusion, group leaders conducted interviews to assess participants’ eligibility according to inclusion and exclusion criteria. Shortly (0-2 weeks) before treatment start a research coordinator organized pre-assessments, immediately followed by randomization. Pre-assessments included participants’ characteristics and outcome measures (the BADS-SF, PPS, ASRS, BADDS, AAQoL, PHQ-9, WHODAS, and the MAAS). Random assignment was performed in blocks of three, assigning two participants to CADDI and one to Hesslinger in each block using a 2:1 ratio. Each clinic had a unique randomization sequence that was transferred to envelopes with serial numbers. After completing the pre-assessment, the research coordinator opened the envelope and communicated the assigned allocation (CADDI or Hesslinger protocol). During treatment, the main outcome measures (BADS-SF, PPS) were rated at sessions 4, 7, and 10 to capture the time trend in changes on these measures through the treatment period. After treatment completion, the research coordinator or group leader administered post-assessments, including outcome measures (BADS-SF, PPS, ASRS, BADDS, AAQoL, PHQ-9, WHODAS, MAAS), participants’ characteristics and a measure of client satisfaction (CSQ-8). Data on outcome measures were collected using pen and paper or online via the Research Electronic Data Capture (REDCap; 57, 58). However, the first group of nine participants was assessed on the BADDS by a blinded clinician pre- and post-treatment. This method was not feasible in all clinical settings due to resource demands and was replaced by the self-rating version of the measure.

### Data analysis

Statistical analyses were performed using SPSS (Version 29, SPSS Inc., Chicago, IL). Treatment effects were analyzed using multilevel modeling to estimate the effects of time and of time by group on continuous outcome measures from the pre-treatment to the post-treatment assessment. Multilevel models are tolerant to heterogeneity and different sample sizes between groups. Models were built stepwise, starting with a basic model with a fixed intercept, then adding random parameters (intercept and slope), and finally adding a time by condition interaction predictor to the model. The maximum likelihood method was used to estimate model parameters, and the models were run using various covariance structures (variance components, unstructured, first order autoregressive, diagonal). Each model’s fit to observed data was evaluated using the -2 log likelihood ratio with significance level set at 0.05. A model with significantly better fit than a previous model was retained. Standardized effect size for between-group effects was calculated using Cohen’s *d* for multilevel models by the formula provided in Feingold (59), using the pooled standard deviation at pre assessment. Effect sizes for within-group effects were calculated with the formula mentioned above, using the standard deviation for each group at pre-assessment. Data were analyzed following the principle of intention-to-treat, using data from all participants included in the trial in the analysis, as multilevel models estimate values that are lost to follow up using available data. Treatment response was assessed using the reliable change index (RCI; Jacobson and Truax (60). RCI was calculated using internal consistency of the outcome measures and the sample standard deviation at pre-treatment, following recommendations in Lambert and Ogles (61). Data of this trial are reported according to the recommendations in the CONSORT statement (62).

## Result

### Between-group effects

Observed means and standard deviations at pre and post assessment are presented in **Table 3**. A significant between-group effect was observed on the BADS-SF, showing a greater improvement in the CADDI group as the model including random intercept, slope, and a time by condition interaction term provided the best fit on the total scale, *F* (1, 110.58) = 4.13, *p* = .045, *d* = 0.49 [0.01, 0.96], and the subscale Activation *F* (1, 89.68) = 6.77, *p* = .011, *d* = 0.65 [0.15, 1.14]. The Avoidance subscale of BADS-SF and the PPS (procrastination) did not show between-groups effect as models with random intercept and slope were not improved by adding a time by condition interaction term. Regarding the secondary outcome measures of ASRS, BADDS, AAQoL and WHODAS, models including random intercept and slope provided the best fit for the total of these scales; adding a time by condition interaction term did not improve fit. This indicates that there were no significant differences between groups on these measures, but there were significant effects of time for both groups on ASRS, BADDS, and AAQoL. Models including a random intercept only provided the best fit on PHQ- 9 and MAAS. For detailed results on the outcome measures, see **Table 4**.

**Table 3:** Observed means and standard deviations of outcome measures at pre and post assessment.

Measure		*n*	Group	Pre-treatment M (SD)	*n*	Post-treatment M (SD)
BADS-SF	Total score	71	CADDI	22.56 (7.30)	52	29.08 (8.11)
		36	Hesslinger	22.36 (10.04)	27	26.52 (10.68)
	Activation	71	CADDI	11.31 (4.26)	52	15.56 (4.52)
		36	Hesslinger	12.25 (5.80)	27	14.19 (5.86)
	Avoidance	71	CADDI	12.75 (4.83)	52	10.48 (5.04)
		36	Hesslinger	13.89 (5.68)	27	11.67 (5.96)
PPS		71	CADDI	45.96 (7.90)	54	39.32 (9.24)
		37	Hesslinger	47.97 (9.44)	26	43.23 (10.69)
ASRS	Total score	69	CADDI	37.51 (8.83)	54	31.69 (10.05)
		37	Hesslinger	39.76 (9.73)	27	33.70 (10.98)
	Inattention	70	CADDI	24.59 (4.74)	54	19.56 (5.50)
		37	Hesslinger	24.57 (4.81)	27	20.63 (6.09)
	Hyperactivity	70	CADDI	13.11 (6.01)	55	12.04 (6.01)
		37	Hesslinger	15.19 (6.66)	27	13.07 (6.84)
BADDS	Total score	70	CADDI	76.51 (14.01)	50	64.46 (19.00)
		34	Hesslinger	81.00 (18.15)	26	70.00 (19.71)
	Organizing and activating to work	71	CADDI	20.07 (3.71)	51	16.37 (5.11)
		35	Hesslinger	20.77 (3.52)	26	17.89 (4.90)
	Sustaining attention and concentration	71	CADDI	18.47 (4.29)	53	15.66 (5.43)
		36	Hesslinger	19.67 (4.33)	27	17.07 (5.61)
	Sustaining energy and effort	71	CADDI	16.93 (4.12)	53	13.96 (4.60)
		36	Hesslinger	18.53 (5.24)	27	16.59 (4.94)
	Managing affective interference	70	CADDI	10.79 (3.94)	52	9.69 (4.18)
		37	Hesslinger	10.92 (4.07)	27	9.19 (3.87)
	Utilizing working memory and accessing recall	71	CADDI	10.42 (3.68)	53	8.85 (3.98)
		36	Hesslinger	11.19 (3.74)	27	9.67 (3.56)
AAQoL	Total score	71	CADDI	44.46 (11.33)	53	55.65 (13.70)
		37	Hesslinger	43.25 (14.92)	28	51.51 (12.69)
	Productivity	71	CADDI	41.17 (14.58)	53	55.36 (16.49)
		37	Hesslinger	38.94 (15.20)	28	48.21 (12.10)
	Relationships	71	CADDI	54.16 (21.20)	53	62.12 (16.55)
		37	Hesslinger	52.30 (25.38)	28	62.23 (20.21)
	Psychological Health	71	CADDI	42.49 (18.16)	53	51.10 (16.97)
		37	Hesslinger	43.13 (19.67)	28	47.32 (17.97)
	Life outlook	71	CADDI	42.45 (13.33)	53	55.55 (16.40)
		37	Hesslinger	43.49 (14.72)	28	52.37 (14.86)
PHQ-9		71	CADDI	11.56 (5.35)	55	9.04 (5.39)
		37	Hesslinger	11.65 (6.65)	28	9.21 (5.66)
WHODAS		71	CADDI	14.47 (7.43)	55	13.75 (8.33)
		37	Hesslinger	13.00 (7.20)	27	12.15 (7.19)
MAAS		71	CADDI	3.61 (0.82)	55	3.83 (0.73)
		37	Hesslinger	3.59 (0.83)	27	3.80 (0.69)
CSQ			CADDI	Not assessed	55	27.31 (4.27)
			Hesslinger	Not assessed	25	24.32 (3.44)

BADS-SF, Behavioral Activation for Depression Scale; PPS, Pure Procrastination Scale; ASRS, Adult ADHD Self report Scale; BADDS, Brown Attention -Deficit Disorder Scales; AAQoL, Adult ADHD Quality of Life Questionnaire; PHQ-9, Patient Health Questionnaire-9; WHODAS, World Health Organization Disability Assessment Schedule; MAAS, Mindful Attention Awareness Scale; CSQ, Client Satisfaction Questionnaire.

**Table 4:** Statistics for between- group analysis.

	Model*	*F*	*df*	*p*	*d*	95% CI
BADS-SF, total	3	4.13	1, 110.58	.045	0.49	[0.01, 0.96]
Activation	3	6.77	1, 89.68	.011	0.65	[0.15, 1.14]
Avoidance	2	10.46	1, 89.86	.002	-0.33	[-0.53, -0.14]
PPS	2	37.99	1, 91.19	<.001	-0.64	[-0.84 -0.43]
BADDS, total	2	32.18	1, 78.46	<.001	-0.77	[-1.04, -0.50]
Organizing and activating to work	2	46.29	1, 77.65	<.001	-0.93	[-1.20, -0.66]
Sustaining attention, concentration	2	23.07	1, 82.74	<.001	-0.66	[-0.93, -0.39]
Sustaining energy and effort	3	3.08	1, 85.69	.083	-0.39	[-0.84, 0.05]
Managing affective interference	2	13.00	1, 83.22	<.001	-0.39	[-0.60, -0.17]
Working memory	2	12.84	1, 86.13	<.001	-0.39	[-0.61, -0.17]
ASRS, total	2	45.65	1, 82.12	<.001	-0.64	[-0.83, -0.45]
Inattentive	2	52.08	1, 87.48	<.001	-0.98	[-1.26, -0.71]
Hyperactive/Impulsive	2	10.03	1, 83.09	.002	-0.20	[-0.32, -0.07]
PHQ-9	1.5	20.92	1, 88.37	<.001	-0.42	[-0.61, -0.24]
MAAS	1.5	6.79	1, 88.00	.011	0.26	[0.06, 0.45]
WHODAS	2	2.47	1, 85.59	.120	-0.15	[-0.34, 0.04]
AAQoL, total	2	62.22	1, 85.68	<.001	0.79	[0.59, 0.98]
Life productivity	3	4.64	1, 85.09	.034	0.45	[0.04, 0.87]
Life outlook	2	49.78	1, 86.71	<.001	0.81	[0.58, 1.04]
Psych health	1.5	16.76	1, 85.81	<.001	0.39	[0.20, 0.57]
Relationships	1.5	19.32	1, 86.91	<.001	0.39	[0.21, 0.56]

BADS-SF, Behavioral Activation for Depression Scale Short Form; PPS, Pure Procrastination Scale; ASRS, Adult ADHD Self report Scale; BADDS, Brown ADD Scales; MAAS, Mindful Attention Awareness Scale; WHODAS, World Health Organization Disability Assessment Schedule; PHQ-9, Patient Health Questionnaire-9; AAQoL, Adult ADHD Quality of Life Questionnaire.

*Model 3 = Statistics for effects of the group x time interaction, i.e. between-group effects, model 2 = Statistics for effects of time, with no group interaction, model 1,5 = Statistics for effects of time using a random intercepts model.

### Within-group effects and treatment response

Overall, within-group effect sizes were larger, on several measures twice as large, in the CADDI condition relative to the control condition. Generally, effect sizes were more often in the large realm for the CADDI condition while the control condition showed moderate effect sizes on several outcome measures (BADS-SF, PPS, BADDS, AAQoL). See **Table 5** for details regarding within-groups effects.

**Table 5:** Within-group effects of outcome measures in each group.

	Condition	F	*df*	*p*	*d*	95% CI
BADS-SF, total	CADDI	32.47	1, 73.16	<.001	0.81	[0.53, 1.09]
	Hesslinger	0.69	1, 28.91	.414	0.17	[-0.25, 0.58]
Activation	CADDI	46.04	1, 82.54	<.001	0.97	[0.69, 1.25]
	Hesslinger	0.65	1, 32.75	.427	0.17	[-0.26, 0.60
Avoidance	CADDI	9.64	1, 60.15	.003	-0.40	[-0.66, -0.14]
	Hesslinger	1.68	1, 29.72	.205	-0.21	[-0.54, 0.12]
PPS	CADDI	31.81	1, 60.72	<.001	-0.79	[-1.06, -0.51]
	Hesslinger	7.70	1, 30.97	.009	-0.42	[-0.73, -0.11]
ASRS, total	CADDI	33.97	1, 54.46	<.001	-0.69	[-0.92, -0.45]
	Hesslinger	12.30	1, 27.74	.002	-0.56	[-0.88, -0.23]
Inattentive	CADDI	43.15	1, 58.42	<.001	-1.08	[-1.41, -0.75]
	Hesslinger	10.86	1, 28.80	.003	-0.77	[-1.25, -0.29]
Hyperactivity/ Impulsivity	CADDI	4.41	1, 55.34	.040	-0.16	[-0.30, 0.01]
	Hesslinger	6.01	1, 27.64	.021	-0.27	[-0.50, -0.04]
BADDS, total	CADDI	23.64	1, 51.87	<.001	-0.94	[-1.33, -0.55]
	Hesslinger	7.21	1, 26.66	.012	-0.46	[-0.81, -0.11]
Organizing and activating to work	CADDI	42.58	1, 51.28	<.001	-1.08	[-1.41, -0.75]
	Hesslinger	7.81	1, 26.32	.010	-0.63	[-1.10, -0.17]
Sustaining attention and concentration	CADDI	15.27	1, 56.22	<.001	-0.70	[-1.06, -0.34]
	Hesslinger	6.88	1, 27.04	.014	-0.50	[-0.90, -0.11]
Sustaining energy and effort	CADDI	24.67	1, 57.29	<.001	-0.74	[-1.03, -0.44]
	Hesslinger	2.15	1, 28.55	.153	-0.21	[-0.50, 0.08]
Managing affective interference	CADDI	8.96	1, 52.98	.004	-0.40	[-0.67, -0.13]
	Hesslinger	4.37	1, 30.64	.045	-0.37	[-0.73, -0.04]
Utilizing working memory and recall	CADDI	9.03	1, 58.22	.004	-0.43	[-0.72, -0.15]
	Hesslinger	3.53	1, 27.99	.71	-0.29	[-0.60, 0.00]
AAQoL	CADDI	50.85	1, 54.96	<.001	1.00	[0.72, 1.29]
	Hesslinger	13.28	1, 29.38	.001	0.43	[0.19, 0.68]
Life productivity	CADDI	53.21	1, 54.44	<.001	0.98	[0.71, 1.30]
	Hesslinger	11.51	1, 54.44	.002	0.49	[0.20, 0.79]
Life outlook	CADDI	43.64	1, 55.06	<.001	0.96	[0.67, 1,25]
	Hesslinger	9.56	1, 63	.003	0.54	[0.19, 0.88]
Psych health	CADDI	16.43	1, 55.74	<.001	0.51	[0.26, 0.77]
	Hesslinger	1,19	1, 29.30	.285	0.13	[-0.11, 0.71]
Relationships	CADDI	10.59	1, 56.15	.002	0.40	[0.15, 0.65]
	Hesslinger	8.32	1, 29.88	.007	0.34	[0.10, 0.57]
PHQ-9	CADDI	20.47	1, 58.15	<.001	-0.50	[-0.74, -0.28]
	Hesslinger	3.43	1, 29.02	.074	-0.29	[-0.61, 0.03]
WHODAS	CADDI	3.97	1, 56.15	.051	-0.23	[-0.45, 0.00]
	Hesslinger	0.07	1, 28.88	.801	-0.05	[-0.41, 0.32]
MAAS	CADDI	3.61	1, 60.86	.062	0.24	[-0.01, 0.50]
	Hesslinger	4.40	1, 28.94	.045	0.30	[0.01, 0.58]

BADS-SF, Behavioral Activation for Depression Scale; PPS, Pure Procrastination Scale; ASRS, Adult ADHD Self report Scale; BADDS, Brown Attention -Deficit Disorder Scales; AAQoL, Adult ADHD Quality of Life Questionnaire; PHQ-9 Patient Health Questionnaire-9; WHODAS, World Health Organization Disability Assessment Schedule; MAAS, Mindful Attention Awareness Scale.

Considering treatment response using reliable change index (RCI), there was a similar trend with a larger proportion of participants in the CADDI group showing to be treatment responders. Although there in some measures were twice as many responders in the CADDI group this trend was not significant for any of the measures. For details see **Table 6**.

**Table 6:** Reliable change in outcome measures of CADDI and Hesslinger.

Measure	Group	*n*	N (%) improved	χ^2^ improved	*p*	N (%) deteriorated
BADS-SF	CADDI	71	19 (26.8)	χ^2^ = 2.28	.150	0
	Hesslinger	36	5 (14.0)			1 (2.8)
PPS	CADDI	71	21 (29.6)	χ^2^ = 1.44	.257	2 (2.8)
	Hesslinger	37	7 (18.9)			0
ASRS	CADDI	69	15 (21.7)	χ^2^ = 0.93	. 437	2 (2.9)
	Hesslinger	37	5 (13.5)			0
BADDS	CADDI	70	24 (34.3)	χ^2^ = 3.75	.070	2 (2.9)
	Hesslinger	34	6 (17.7)			0
AAQoL	CADDI	71	24 (33.8)	χ^2^ = 3.75	.070	1 (1.4)
	Hesslinger	37	6 (16.2)			0
PHQ-9	CADDI	71	18 (25.4)	χ^2^ = 2.03	.216	1 (1.4)
	Hesslinger	37	5 (13.5)			0
WHODAS	CADDI	71	7 (9.9)	χ^2^ = 1.82	.259	3 (4.2)
	Hesslinger	37	1 (2.7)			4 (10.8)
MAAS	CADDI	71	8 (11.3)	χ^2^ = 0.12	.761	4 (5.6)
	Hesslinger	37	5 (13.5)			0

BADS-SF, Behavioral Activation for Depression Scale; PPS, Pure Procrastination Scale; ASRS, Adult ADHD Self report Scale; Brown ADD Scale, AAQoL, Adult ADHD Quality of Life Questionnaire; PHQ-9 Patient Health Questionnaire; WHODAS, WHO Disability Assessment Schedule; MAAS, Mindful Attention Awareness Scale.

### Other measures

Considering adherence to treatment, on average participants attended 9.56 (SD = 3.92) sessions in the intervention group and 10.59 (SD = 3.89) in the control group. Participants received a mean of 8.91 (SD = 3.33) weekly telephone calls in the intervention group and 8.14 (3.44) in the control group. Sixteen participants (22.5%) dropped out of the CADDI group and seven participants (21.3%) dropped out of the Hesslinger group. None of these differences between groups were significant when analyzed with t-tests and chi-2 tests. Adherence to home assignments, however, showed no difference between groups at session 4 but differed at session 7; *t* (32.08) = 2.94, *p* = 0.006 and 10; *t* (37.67) = 2.28, *p* = 0.013 with a significantly larger adherence in the CADDI group. For further details on adherence to treatment see **Table 7**. Dropouts were analyzed using Pearson’s chi-square tests, showing no significant differences in proportions between dropouts and completers regarding gender, medication status, education, occupation and income at pre-assessment.

**Table 7:** Participants’ adherence to allocated intervention.

Adherence to treatment		CADDI (*n*= 71)	Hesslinger (*n*=37)
		M (SD)	M (SD)
Sessions attendance		9.56 (3.92)	10.59 (3.89)
Phone calls		8.91 (3.33)	8.14 (3.44)
Compliance to home Assignments			
	Week 4^a,b^	4.25 (0.69)	4.07 (1.36)
	Week 7^c,d^	4.13 (0.83)	3.20 (1.47)
	Week 10^e,f^	4.23 (0.78)	3.65 (1.16)

^a^Missing: CADDI, *n* = 4, ^b^Missing: Hesslinger, *n* = 8, ^c^Missing: CADDI, *n* = 6, ^d^Missing: Hesslinger, *n* = 12, ^e^Missing: CADDI, *n* = 8, ^f^Missing: Hesslinger, *n* = 11.

Therapists’ adherence to the treatment protocol were generally good in both conditions, the mean values of the nine item ranged from 3.40 to 3.85 (response scale ranging from 0 to 4) and showed no difference between groups at session 4, but at session 7 there was a significantly higher adherence in therapists in the CADDI condition (M = 3.85, SD = 0.14) compared to the Hesslinger condition (M = 3.52, SD= 0.34), *t* (18) = 2.58, *p* = .019. Similarly, at session 10 therapists in the CADDI condition rated their adherence significantly higher (M = 3.67, SD = 0.31) than therapists in the Hesslinger condition (M = 3.40, SD = 0.26), *t* (12) = 2.43, *p* = .024.

Participants’ treatment satisfaction as measured using the CSQ-8 was significantly higher in the CADDI condition (M = 27.31, SD=4.27) compared to the Hesslinger condition (M = 24.32, SD = 3.44), *t* (57.05) = 3.33, *p* = 0.002. Therapists in the CADDI condition rated the usefulness of the protocol as significantly greater (M = 3.50, SD= 0.43) than therapists in the Hesslinger condition (M = 2.14, SD = 0.39), *t* (12.81) = 7.58 *p* = <.001.

## Discussion

The primary aim of this study was to investigate the effectiveness of CADDI, a newly developed CBT protocol for ADHD-I. CADDI introduces new features, including strategies and skills training focused on organization and activation, combined with mindfulness. In this RCT, the CADDI protocol was compared to standard ADHD psychological treatment, the Hesslinger protocol, to assess whether CADDI, with its focus on organization and activation, was more effective for ADHD-I. As hypothesized, participants randomized to the CADDI group showed significantly greater improvement on the primary outcome of activation, as measured by the BADS-SF, supporting the efficacy of this intervention. Although there was no significant between-group effect on the other primary outcome of procrastination, as measured by the PPS, within-group analysis showed a much larger effect size in the CADDI group, suggesting that the sample size might have been too small to detect a between-group effect. Furthermore, RCI revealed that more participants in the CADDI group experienced a reliable change in PPS. Because the focus on activation is a new component in the treatment of ADHD, there are few studies for comparison regarding its effects in this patient group. However, in a study by Oddo et al. (63) BA significantly increased goal directed behavior in a group of at-risk student drinkers with ADHD, thereby decreasing negative outcomes of drinking. Furthermore, Fernández-Rodríguez et al. (64) showed BA to be the most efficacious condition in transdiagnostic treatment of anxiety and depression as compared to other CBT protocols.

Regarding secondary outcomes there were no significant between-group effects in the MLM analysis. Compared to the control group though, CADDI participants showed a better response pattern on the majority of secondary outcome measures. This trend was most noticeable on the BADDS and AAQoL, where the total scale showed effect sizes twice as large for the CADDI group. As hypothesized, measures of ADHD symptoms did not show significant between-group effects. Instead, the ASRS showed medium to large effect sizes for both groups. Moreover, the ASRS demonstrated much larger effects on inattention compared to hyperactivity, which aligns with previous observations (23). The within- group effect sizes on symptoms of inattention, (*d =* -1.08 for CADDI and *d =* -0.77 for Hesslinger) can be compared to the standard mean difference of 0.53 calculated for inattention in a meta-analysis across various CBT interventions (14). The small effect sizes on hyperactivity likely reflect that the participants had low symptom levels at the pre-assessment and thereby little room for improvement. Furthermore, our results on reducing ADHD symptoms are comparable to, and in some cases better than, those reported in other studies (11). Comparing to other studies of the Hesslinger protocol, effect sizes for ADHD symptoms observed by Morgensterns et al. (19) and Hirvikoski et al. (18) were equivalent to results in this present study.

The MAAS showed small effects in both groups, however the within group analysis showed a significant effect of time for the Hesslinger group only. This might be explained by the greater focus on mindfulness in the Hesslinger protocol. Although mindfulness practice is an important component in both protocols, effect sizes on MAAS were small and a similar finding was made in the study by Morgensterns et al. (19). However, mindfulness practice has been associated with decrease in ADHD symptoms and executive dysfunction in several studies investigating the effects of mindfulness practice on ADHD symptom (41, 44). The small effect sizes in this present study might be due to observed means at pre assessment being close to means observed in a non-clinical group of university students (55) and means at post assessment are even closer to that of the students. Self-ratings of mindful awareness though, could possibly be challenging to individuals who habitually are absentminded, and one effect of treatment is a greater awareness of what is occupying attention. After treatment participants may be more observant of their absentmindedness and thereby not perceiving nor reporting much improvement on the MAAS. Interestingly, in the interview study (38) a distinct result was that participants experienced increased awareness after treatment and expressed an unanimous appreciation of mindfulness practice. The lack of observed effects on functional impairment, as measured by WHODAS, may be because the WHODAS includes areas of functionality related to physical health, which were not expected to be influenced by either intervention.

In terms of adherence, both interventions performed well regarding attrition and session attendance, indicating that both interventions were appreciated and worthwhile for participants. When compared to attrition rates reported in other studies of group CBT for ADHD, which range from 20% to 32.8% (18, 65, 66), this study performed in the lower range. This attrition rate was accomplished despite pandemic-related dropouts, which accounted for at least five participants (20% of the total dropouts), suggesting that adherence rates could have been even better under different circumstances. The attrition rate in this study was notably lower than the 33.3% reported in the feasibility study (37). Due to the less satisfactory results in the feasibility study, a change was made in the therapy’s administration to include weekly individual telephone support throughout the entire treatment period. Other studies (67, 68), have also used a combination of group and individual coaching for ADHD, while others have not (18, 20). Participants in the interview study of CADDI expressed that the phone calls were a valuable aid in maintaining progress in therapy (38), leading us to suggest that individual support, in addition to group therapy, may be an important adaptation of CBT for individuals with ADHD.

Regarding patient satisfaction, treatment in the CADDI protocol was perceived significantly more positive than in the Hesslinger protocol. Therapist ratings echoed this sentiment, with a more favorable evaluation of CADDI for treating ADHD-I. This may be due the CADDI protocol being designed specifically for patients with ADHD-I, including components tailored to address the most prominent problem areas in this specific ADHD presentation.

### Strengths and limitations

To our knowledge, CADDI is the first CBT protocol specifically designed for the inattentive presentation of ADHD and the first to incorporate behavioral activation and procrastination strategies—areas relevant for increasing quality of life in ADHD-I. Additionally, this study appears to be the first to compare the effects of two psychological treatments for adult ADHD. This multicenter study was conducted in regular psychiatric outpatient care at six different clinics, representing a variety of socioeconomic conditions. Moreover, generalizability to other outpatient psychiatric care settings was strengthened by the recruitment of participants and therapists from routine care, along with minimal exclusion criteria. The sample reflects the population of therapy-seeking ADHD-I patients, who are more likely to be women, diagnosed with ADHD in adulthood and previously treated for anxiety and depression, as reported by Siddiqui et al. (69). Further, the control condition in this study well represents treatment as usual, as the Hesslinger protocol is typically offered in groups composed of individuals with various ADHD presentations. Thus, the setting and recruitment procedure provided grounds for assuming good external validity. Further, the therapists were supervised by clinical experts in the protocols and thereby adherence to treatment protocol was reinforced while also providing therapists with support and guidance on matters arising in the groups.

This study had several limitations that should be considered. First, the study lacked clinician-rated assessment of outcomes, increasing the risk of expectation bias to influence the results. Blinded, clinician-rated BADDS was originally meant to be included but had to be replaced with self-ratings due to resource and funding constraints. Although a blinded clinicians-rated assessment would have been preferable, self-ratings of ADHD symptoms have been shown to be reliable (70). Second, the trial was underpowered as the intended sample size of 144 participants could not be reached, due to restrictions during the pandemic, and in its aftermath, due to restraints in resources the pandemic caused in clinical care. The study failed to show significant between-group effects on several measures (AAqoL, BADDS, PPS), despite large differences in within-group effect sizes on these measures, indicating lack of power in the sample. Further, the uneven group sizes may contribute to a larger uncertainty in the within-group analysis of the smaller group. Consequently, the potentially stronger effect of CADDI on measures of procrastination and quality of life, remains inconclusive and warrants further investigation. Third, the study did not assess therapists’ adherence to treatment protocols by other measures than self-ratings.

### Conclusions and implications for the clinic

This trial demonstrated that CADDI, the first CBT protocol specifically tailored to individuals with ADHD-I, was more effective on the primary outcome measure of behavioral activation compared to the Hesslinger CBT treatment. Additionally, results indicated that participants in the CADDI group showed larger effect sizes on measures of procrastination, symptoms of inattention as measured by the BADDS, depression, and quality of life compared to the active control condition. Both participants and therapists also reported higher satisfaction with the CADDI protocol. This study highlights the potential of incorporating strategies from behavioral activation in the treatment of initiating difficulties in ADHD-I, and this approach warrants further attention. Furthermore, this trial shows that passivity in ADHD-I can be effectively treated and suggests a potential advantage of an intervention specifically targeting ADHD-I over generic CBT for ADHD.

Additionally, the study supports the beneficial effects of the Hesslinger protocol for ADHD-I, as demonstrated in previous studies. Both group treatments were appreciated and can be considered viable treatment options for ADHD-I, given that adequate individual support is provided. Our results underline the importance of attending to the individual while offering group interventions, and the combination gives the benefits of the group while also providing opportunity to adapt treatment to everyone’s needs. Conclusively, the findings highlight the promise of CADDI as an ADHD-I-specific intervention, while emphasizing the need for future research with larger sample sizes and long-term follow-ups to validate and expand upon these results.

## Data Availability

The datasets presented in this article are not readily available because data cannot be shared publicly because of Swedish legal and ethical restrictions related to sensitive patient data information. Specifically, the participants did not consent to public data sharing. Data are available from Region Stockholm (contact via data protection officer Camilla Heise Löwgren, camilla.heise-lowgren@regionstockholm.se) for researchers who meet the criteria for access to confidential data. Requests to access the datasets should be directed to Camilla Heise Löwgren, camilla.heise-lowgren@regionstockholm.se.
